# Diabetes and Cancer: Epidemiological, Clinical, and Experimental Perspectives

**DOI:** 10.1155/2012/101802

**Published:** 2012-10-02

**Authors:** Chin-Hsiao Tseng, Chien-Jen Chen, Joseph R. Landolph

**Affiliations:** ^1^Department of Internal Medicine, National Taiwan University College of Medicine, Taipei 100, Taiwan; ^2^Genomics Research Center, Academia Sinica, Taipei 115, Taiwan; ^3^Cancer Research Laboratory, USC/Norris Comprehensive Cancer Center, and Depatrments of Molecular Microbiology/Immunology, and Pathology, Keck School of Medicine, University of Southern California, Los Angeles, CA 90033, USA; ^4^Department of Molecular Pharmacology/Pharmaceutical Sciences, School of Pharmacy, University of Southern California, Los Angeles, CA 90033, USA

Diabetes is a major cause of death in many countries due to its increasing incidence, high prevalence, and clinical manifestation of a variety of micro- and macrovascular complications if it is not appropriately treated [1, 2]. Recent studies have shown that diabetic patients may also have a higher risk of cancer [2], the number one killer that threatens the lives of billions of people. 

The clarification of the link between these two important diseases, new developments in clinical technologies and medications for the management and improvement of the survival of cancer patients in diabetic and nondiabetic individuals, and the understanding of the basic mechanisms of cancer and diabetes and their interrelationships are urgently needed for the prevention of these two diseases and the provision of better care to the patients. 

In this special issue, original as well as review articles on the relationship between diabetes and cancer from the epidemiological, clinical, and experimental perspectives were invited and called for. We accepted ten papers for the publication of this special issue after careful consideration of a wide spectrum of factors including originality, novelty, and potential impacts.

In a research article, by using the National Health Insurance database from Taiwan, M. C. Hsieh et al. demonstrated a significantly higher risk of cancers involving the breast, prostate, colon, lung, liver, and pancreas in the diabetic patients. Furthermore, they disclosed that patients using insulin or sulfonylureas were more likely to develop cancer than those who used metformin. The significantly higher risk of prostate cancer associated with diabetes in the Taiwanese population in this study is consistent with a previously published paper from Taiwan [3], but is contrary to a significantly lower risk observed in Caucasian people [4]. Such a discrepancy between different ethnicities awaits further exploration.

According to the review article by N. Hara (Niigata University, Niigata, Japan), diabetes is associated with higher incidences of many cancers, including lung cancer, stomach cancer, colorectal cancer, liver cancer, and pancreatic cancer. In addition, diabetic patients may have a lower risk of overall prostate cancer, but the risk of advanced high-grade prostate cancer is actually higher in diabetic patients. Lower levels of testosterone and prostate-specific antigen observed in the diabetic patients might help explain the high-grade prostate cancer in these patients. Furthermore, androgen-deprivation therapy used for the treatment of prostate cancer may also induce insulin resistance and diabetes. 

One of the mechanisms explaining the higher risk of cancer in the diabetic patients is insulin resistance. In this special issue, B. Arcidiacono et al. (Magna Græcia University of Catanzaro, Catanzaro, Italy) gave a thorough overview on the pathogenetic mechanisms linking insulin resistance and cancer risk.

The association between diabetes and thyroid cancer has been rarely studied. S. R. Shih et al. from Taiwan wrote a nice review of the literature on this topic and proposed some potential mechanisms linking diabetes and thyroid cancer. However, future studies are required to confirm these new hypotheses. W. Y. Chiu et al. reviewed the most updated literature regarding the potential risk of thyroid cancer induced by the newly launched antidiabetic medication of glucagon-like peptide-1 receptor agonists, including exenatide and liraglutide. It is worthwhile to note that the risk may not be limited to the rare form of medullary thyroid cancer, but may also involve the more commonly seen papillary thyroid cancer.

Whether glycemic control may affect the outcome of patients with cancer is still under debate. The retrospective analyses of 265 patients with advanced breast cancer by C. Villarreal-Garza et al. from Mexico suggested that glycemic control may be an important factor related to survival in either the diabetic or nondiabetic patients. A level of >130 mg/dL is associated with a significantly higher risk of mortality.

The association between diabetes and pancreatic cancer has long been observed [5]. However, whether diabetes may induce pancreatic cancer has always been challenged because diabetes is always newonset when a diagnosis of pancreatic cancer follows [6]. A recent study suggested that new-onset diabetes with a history of dyslipidemia may predict a higher risk of pancreatic cancer [6]. The retrospective study by J. Trna et al. from Czech Republic, which evaluated the autopsy reports of 182 pancreatic cancer patients and 135 control subjects without pancreatic cancer, suggested a predominance of female sex among pancreatic cancer patients with diabetes. Although the result is preliminary, this study provided a hint for a future look into the sexual discrepancy in the risk of diabetes-related pancreatic cancer. The study by H. Yu et al. from China suggested that elevated tumor-associated antigen CA19-9 from pancreatic ductal cells may be associated with high serum total cholesterol level, impaired insulin secretion, and hyperglycemia in the diabetic patients. The findings suggest a close link between pancreatic exocrine and endocrine dysfunction and imply a potential improvement in beta-cell function with the treatment of elevated cholesterol level. 

S. H. Liu and L. T. Lee from the Industrial Technology Institute, Hsinchu, Taiwan demonstrated a two-step differentiation protocol to induce mouse embryonic stem (ES) cells to differentiate into insulin-producing cells. They first treated mouse ES cells with activin to induce the mouse ES cells to differentiate into endodermal cells in a monolayer. Then, they showed that addition of nicotinamide, insulin, and laminin to the endodermal cells induced the endodermal cells to differentiate into insulin-producing cells. In a related study, G. Qing-Song et al. from Nantong University in Nantong, in China, reported a procedure for induction of insulin-producing cells from bone marrow mesenchymal stem cells (MSCs) from mice. G. Qing-Song et al. utilized a novel strategy in which multiple transcription factors—PDX-1, NeuroD1, and MafA—were transfected into mouse MSCs, and this resulted in formation of insulin-producing cells. Both the studies of S. H. Liu and L. T. Lee and the studies of G. Qing-Song et al. pave the way for more efficient beta-cell recruitment for transplantation.

In summary, the link between diabetes and cancer is an interesting issue that requires intensive exploration on different aspects including epidemiological, clinical, and experimental studies. This special issue provides a platform for the publication of some important ongoing researches and concepts. We hope that it can trigger the explosion of more fruitful progress in related fields.

*Chin-Hsiao Tseng* *Chin-Hsiao Tseng* *Chien-Jen Chen* *Chien-Jen Chen* *Joseph R. Landolph Jr.* *Joseph R. Landolph Jr.*
